# Preserved but Less Efficient Control of Response Interference After Unilateral Lesions of the Striatum

**DOI:** 10.3389/fnhum.2018.00414

**Published:** 2018-10-16

**Authors:** Claudia C. Schmidt, David C. Timpert, Isabel Arend, Simone Vossel, Anna Dovern, Jochen Saliger, Hans Karbe, Gereon R. Fink, Avishai Henik, Peter H. Weiss

**Affiliations:** ^1^Cognitive Neuroscience, Institute of Neuroscience and Medicine (INM-3), Research Centre Jülich, Jülich, Germany; ^2^Department of Neurology, University Hospital Cologne, Cologne, Germany; ^3^Department of Psychology and the Zlotowski Center for Neuroscience, Ben-Gurion University of the Negev, Beer-Sheva, Israel; ^4^Department of Psychology, University of Cologne, Cologne, Germany; ^5^Neurological Rehabilitation Centre Godeshöhe, Bonn, Germany

**Keywords:** cognitive control, stroke, simon task, putamen, caudate nucleus

## Abstract

Previous research on the neural basis of cognitive control processes has mainly focused on cortical areas, while the role of subcortical structures in cognitive control is less clear. Models of basal ganglia function as well as clinical studies in neurodegenerative diseases suggest that the striatum (putamen and caudate nucleus) modulates the inhibition of interfering responses and thereby contributes to an important aspect of cognitive control, namely response interference control. To further investigate the putative role of the striatum in the control of response interference, 23 patients with stroke-induced lesions of the striatum and 32 age-matched neurologically healthy controls performed a unimanual version of the Simon task. In the Simon task, the correspondence between stimulus location and response location is manipulated so that control over response interference can be inferred from the reaction time costs in incongruent trials. Results showed that stroke patients responded overall slower and more erroneous than controls. The difference in response times (RTs) between incongruent and congruent trials (known as the Simon effect) was smaller in the ipsilesional/-lateral hemifield, but did not differ significantly between groups. However, in contrast to controls, stroke patients exhibited an abnormally stable Simon effect across the reaction time distribution indicating a reduced efficiency of the inhibition process. Thus, in stroke patients unilateral lesions of the striatum did not significantly impair the general ability to control response interference, but led to less efficient selective inhibition of interfering responses.

## Introduction

Cognitive control refers to a set of cognitive processes implicated in selecting an appropriate task-related action while minimizing interference from possible response alternatives. Therefore, one important aspect of cognitive control is the ability to inhibit prepotent yet inappropriate or interfering response tendencies (Banich, 2009; Goghari and MacDonald, 2009). Two conceptually different components of cognitive control with respect to inhibitory (control) demands are (global) response inhibition and interference control (Nigg, 2000). The former process, *response inhibition*, aims at completely withholding or canceling an inappropriate response, which is usually studied with go/no-go (Falkenstein et al., 1999) or stop-signal tasks (Verbruggen and Logan, 2008). In contrast, *interference control* requires conflict resolution and the selective inhibition of inappropriate response tendencies to enable the execution of the task-appropriate response (Friedman and Miyake, 2004; Diamond, 2013). The current study focusses on response interference control (i.e., the latter component of cognitive control).

A well-established and sensitive measure for the control of response interference is the Simon task (Simon, 1969; Hommel, 2011). In this stimulus-response interference task, participants are instructed to respond to a non-spatial stimulus feature (e.g., color) by giving manual left or right responses, irrespective of the location at which the stimulus appears. In each trial, the stimulus is presented either to the left or to the right of a fixation point. Participants typically respond faster (and more accurate) when the relative spatial location of the stimulus matches the side of the response (congruent condition) compared to when both positions do not correspond (incongruent condition), even though the spatial location of the stimulus is task-irrelevant (Lu and Proctor, 1995). The difference in response times (RTs) between incongruent and congruent conditions is called the Simon effect (Hedge and Marsh, 1975). The magnitude of the Simon effect reflects the extra demand (and thus time) that is required to overcome the stimulus-response interference. Controlling stimulus-response interference in the Simon task is thought to involve selectively inhibiting the prepotent tendency to respond to the (irrelevant) location of the stimulus and instead selecting a task-appropriate response for successful task performance (Burle et al., 2002). The *activation-suppression hypothesis* provides a theoretical framework for the temporal dynamics of the response interference control processes in the Simon task. It proposes that, in incongruent trials, the (incorrect) response tendency as activated by the (task-irrelevant) stimulus location is followed by selective inhibition, which needs some time to develop (Ridderinkhof, 2002). Given these proposed dynamics, the automatic activation of the (incorrect) response prevails in incongruent trials with short reaction times (RTs), while in incongruent trials with long RTs selective inhibition can already exert its effects. Therefore, the integrity (Forstmann et al., 2008) and efficiency (Wylie et al., 2010) of the selective response inhibition process can be revealed by distributional analyses of RTs in which the Simon effect is analyzed as a function of intraindividual response latencies (De Jong et al., 1994; Ridderinkhof et al., 2004b). Specifically, efficient selective inhibition is reflected in a reduced Simon effect as RT increases. Conversely, less efficient selective inhibition rather leads to a uniform or even increased Simon effect across the RT distribution.

It is generally accepted that cortical areas make a fundamental contribution to cognitive control processes, including response interference control (Miller and Cohen, 2001; Nee et al., 2007). In particular, medial and lateral frontal brain regions including the anterior cingulate cortex (ACC) and lateral prefrontal cortex (PFC) are supposed to support the detection and monitoring of (response) conflicts (Botvinick et al., 2004; Kerns, 2006) and the implementation of cognitive control to resolve the conflict (MacDonald et al., 2000; Ridderinkhof et al., 2004a), respectively.

In contrast, the putative contribution of subcortical structures to cognitive control processes, especially to the control of response interference is less clear (O’Callaghan et al., 2014). Models of basal ganglia function suggest that the striatum (comprising the putamen and caudate nucleus) modulates the selection and inhibition of interfering responses via anatomical connections to (pre-) frontal and motor cortical areas (Mink, 1996; Utter and Basso, 2008), thereby potentially contributing to response interference control.

Indeed, clinical observations and studies in patients with neurodegenerative diseases affecting the basal ganglia, such as Parkinson’s disease (PD) and Huntington’s disease (HD), point to a relevant role of the striatum in the control of response interference (Seiss and Praamstra, 2004; Nelson and Kreitzer, 2014). However, previous studies on response interference control in PD and HD patients using the Simon task are far from being conclusive: while some studies reported impairments in resolving response interference in PD or HD patients (Georgiou et al., 1995; Praamstra and Plat, 2001; Fielding et al., 2005; Wylie et al., 2010), others did not find significant differences between patient and control groups (Brown et al., 1993; Cope et al., 1996; Georgiou-Karistianis et al., 2007; Schmiedt-Fehr et al., 2007). These equivocal findings might presumably be due to several experimental variables, including clinical characteristics of the examined PD and HD patients as well as differences in task design and procedures (for a detailed discussion of the potential impact of these diverse factors please refer to Falkenstein et al., 2006; Wylie et al., 2009).

Given the abundant anatomical interconnections between the striatum and (pre-) frontal regions, control of response interference may depend on the integrity of fronto-striatal networks (Liston et al., 2006; Aron et al., 2007; Wiecki and Frank, 2013). Note that the functional (and later structural) alterations in neurodegenerative diseases are not limited to the striatum but extend into (pre-) frontal areas (Reading et al., 2004; Selemon et al., 2004). Therefore, impairments in response interference control in PD and HD patients may reflect abnormal function of the striatum, the (pre-) frontal cortex, or both (Caligiore et al., 2016).

The few available group studies on the impact of stroke-induced striatal lesions on cognitive control processes have revealed deficits in (global) response inhibition using a stop-signal task (Rieger et al., 2003) as well as in cognitive flexibility during task switching (Cools et al., 2006; Yehene et al., 2008). Other studies investigating cognitive control processes after striatal lesions have reported (non-specific) deficits in cognitive control using standard neuropsychological tests (Hochstenbach et al., 1998; Ward et al., 2013). Moreover, single case studies revealed (specific) impairments in response selection, interference control, or cognitive flexibility (Dubois et al., 1995; Swainson and Robbins, 2001; Benke et al., 2003; Rainville et al., 2003). Finally, neuroimaging studies in healthy subjects employing (variants of) the Simon task also revealed involvement of the striatum in controlling response interference (Peterson et al., 2002; Wittfoth et al., 2009).

Accordingly, the present study aimed at further investigating the putative role of the striatum in the control of response interference in 23 patients with unilateral stroke-induced striatal lesions by using the Simon task. This task was chosen because it was commonly adopted in previous studies investigating response interference control processes in neurological patients (e.g., Georgiou-Karistianis et al., 2007; Wylie et al., 2010) and has been shown to engage the striatum (e.g., Peterson et al., 2002; Stocco et al., 2017). The overall Simon effect was used to infer the general ability to control response interference, with larger RT differences (i.e., larger Simon effects) being associated with impaired control of response interference. Further distributional analyses of RTs assessed the efficiency of the selective inhibition process engaged in resolving response interference. Here, a decrease in the magnitude of the Simon effects across the RT distribution reflects efficient selective inhibition of interfering responses (Ridderinkhof, 2002). To the best of our knowledge, no stroke lesion study has yet investigated the specific role of the striatum with respect to the general ability and efficiency of response interference control as measured by the Simon task. If integrity of the striatum is required for the control of response interference, unilateral striatal lesions might impair performance in the Simon task. In particular, when compared to healthy control subjects, stroke patients with striatal involvement could exhibit: (i) an overall larger Simon effect (indicating impaired control of response interference) and/or (ii) less reduction in the Simon effect across the RT distribution (indicating less efficient selective inhibition).

## Materials and Methods

### Participants

A total of 43 patients with first-ever ischemic or hemorrhagic stroke affecting the left or the right hemisphere were consecutively recruited from the Department of Neurology, University Hospital Cologne (*n* = 21) and the Neurological Rehabilitation Centre Godeshöhe, Bonn (*n* = 22). Inclusion criteria were right-handedness, age between 18 and 90 years, no other neurological disorders, no current or previous psychiatric diseases, and no current or history of substance abuse or dependance.

Stroke lesions were identified based on clinical imaging data (computed tomography (CT): *n* = 9, magnetic resonance imaging (MRI): *n* = 34). All lesions were mapped by drawing the lesions manually in steps of 5 mm on axial slices of a T1-weighted template brain (ch2.nii) provided by MRIcron^1^. Lesion mapping was performed by DCT and consecutively checked by AD. Both examiners had to jointly agree upon the exact lesion location and extent and were blind to the individual patient’s task performance at the time of lesion mapping (for further methodological descriptions see also Timpert et al., 2015).

The aim of the current study (i.e., to investigate the role of the striatum in the control of response interference) required that the patients’ unilateral stroke involved the striatum (putamen and/or caudate nucleus). Involvement of the striatum was verified using a mask of the striatum (putamen and caudate nucleus, see Supplementary Figure S1) derived from the Harvard-Oxford atlas of cortical and subcortical structures provided by the Harvard Center for Morphometric Analysis^2^ and distributed with FSL^3^. Accordingly, 19 patients were excluded after enrolment because their clinical imaging did not reveal any striatal involvement. A 20th patient was excluded due to chance-level performance in the Simon task.

Thus, a total of 23 stroke patients (12 female), including nine patients with left hemispheric (LH) and 14 patients with right hemispheric (RH) stroke were included in the subsequent analyses. Twenty patients suffered from an ischemic, three from a hemorrhagic stroke. The mean age was 57.1 years (*SD* = 15.1 years, range 25–84 years). All patients were examined during the sub-acute (i.e., >24 h post-stroke; Hillis et al., 2002) or chronic stage of their disease. The mean time interval between stroke onset and experimental assessment was 76.3 days (*SD* = 30.5 days, range 2–514 days). The time interval did not differ significantly between LH and RH stroke patients (*t*_(21)_ = −0.35, *p* = 0.731). Furthermore, there were no significant correlations between days since onset of stroke and any of the key measures in the Simon task (e.g., overall mean reaction time, mean Simon effects, total error rate; all *p*-values >0.278).

**Figure 1:**
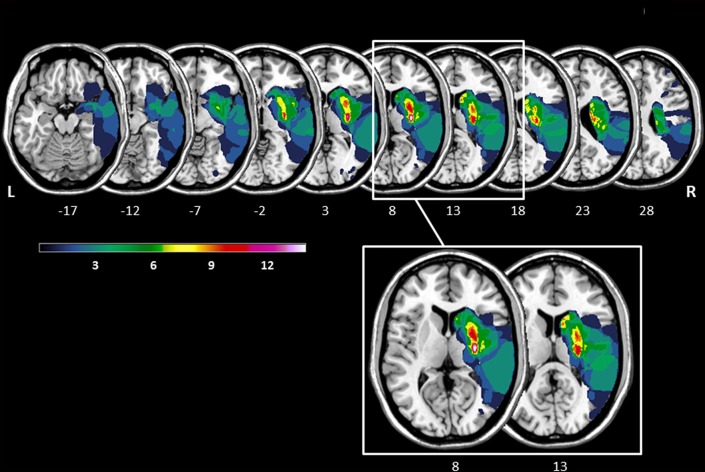
Lesion overlay of the stroke patients with striatal involvement (*n* = 23, left hemisphere stroke: *n* = 9, right hemisphere stroke *n* = 14). All lesions were flipped to the right hemisphere. Color shades represent the increasing number of overlapping lesions (from cold to warm colors). Axial slices with MNI z-coordinates from −17 to 28 are shown. The striatum (putamen and caudate nucleus) is visible in the axial slices with the MNI z-coordinates ranging from −12 to 23 (see Supplementary Figure S1 for a mask of the striatum). Axial slices with the MNI z-coordinates 8 and 13 indicating the highest lesion overlap within the putamen and the head of the caudate nucleus are highlighted. The figure was generated using the freely available MRIcron software package (Rorden and Brett, 2000).

The lesion overlay plot of the 23 stroke patients with striatal involvement is shown in Figure 1. In all patients, the territory of the middle cerebral artery was involved. Consistent with the inclusion criteria, the highest lesion overlap was observed within the putamen and the head of the caudate nucleus. In some patients the lesions also extended to adjacent subcortical (central white matter tracts, globus pallidus, thalamus) and cortical regions (insula, frontal cortex). There was no significant difference between LH and RH stroke patients concerning lesion size (*t*_(21)_ = −1.56, *p* = 0.134), and no significant correlation between number of lesioned voxels and any of the key Simon task measures (all *p*-values > 0.327).

Consistent with the predominantly subcortical lesion pattern, neuropsychological deficits (e.g., neglect, apraxia, aphasia, executive dysfunction) were mild in the current patient sample. The 23 stroke patients did not suffer from unspecific cognitive decline, since all patients performed above the cut-off of 24 out of 30 points of the Mini-Mental Status Examination (MMSE; Folstein et al., 1975). None of the patients showed relevant signs of neglect, apraxia, or aphasia according to the Neglect Test (NET; Fels and Geissner, 1996, i.e., the German version of the Behavioral Inattention Test (BIT), Wilson et al., 1987), the Cologne Apraxia Screening (KAS; Weiss et al., 2013), or the short version of the Aphasia Check List (ACL-K; Kalbe et al., 2002), respectively. There was mild (to moderate) impairment of executive functioning as assessed with the Trail Making Test (TMT; Reitan and Wolfson, 1993) and the Stroop Color-Word Interference Test (SCWT; Bäumler, 1984).

With respect to clinical scores, all patients were rated to be mild to moderately disabled according to the modified Rankin Scale (Rankin, 1957). On average, stroke patients exhibited mild to moderate paresis of the contralesional hand and arm as assessed by the Medical Research Council (MRC) paresis scale (Medical Research Council, 1976). There were no significant differences between LH and RH stroke patients concerning any of the above-mentioned clinical characteristics (all *p*-values > 0.091). The neuropsychological and clinical data of the 23 stroke patients are summarized in Table 1.

**Table 1:** Neuropsychological and clinical data for the stroke patients (*n* = 23).

	Mean	SD	Score range
MMSE	28.4^a^	1.5	25–30
BIT line bisection score	8.8^a^	0.4	8−9
BIT star cancellation LI	0.0^a^	0.0	−0.02–0.06
BIT text reading total words	138.0^b^	4.2	121−140
KAS total	79.7^c^	0.7	78–80
ACL-K total	35.9^b^	3.8	28–40
TMT—Part B/Part A	2.8^c^	1.0	1.5–5.0
SCWT—Interference (sec)	116.5^c^	67.1	54–342
Rankin scale	2.2	1.0	1–4
MRC paresis scale hand	3.6	1.4	0–5
MRC paresis scale arm	3.8	1.2	0–5

*Means, standard deviations (SD) and score ranges are provided. MMSE = Mini-Mental Status Examination (maximum score 30 points; cut-off ≤24 points). BIT = Behavioral Inattention Test (neglect-specific cut-off criteria as defined in Eschenbeck et al. (2010): BIT line bisection test: line bisection score ≤7 points; BIT star cancellation test: laterality index (LI) ≤ −0.2, with LI = (hits left − hits right)/(hits left + hits right); BIT text reading test: neglect of either one left paragraph or of the left words in at least two different lines of a newspaper article arranged in three columns each consisting of two paragraphs (total words: 140); none of the patients showed signs of neglect). KAS = Cologne Apraxia Screening (maximum score 80 points; cut-off ≤76 points). ACL-K = Aphasia Check List-short version (maximum score 40 points; cut-off <33 points; mild (26–32 points), moderate (15–25 points), severe (0–14 points) language impairment). TMT = Trail Making Test (the ratio score Part B/Part A was corrected for age and transformed into *z*-values; the mean ratio score of 2.8 for the stroke patients corresponds to a *z*-value of −0.6). SCWT = Stroop Color-Word Interference Test (the time in seconds in the interference condition was corrected for age and transformed into *T*-values; the mean time in the interference condition of 116.5 scorresponds to a *T*-value of 47). Rankin scale (grades: 0 = No symptoms at all; 1 = No significant disability despite symptoms; 2 = Slight disability; 3 = Moderate disability; 4 = Moderately sever disability; 5 = Severe disability). MRC = Medical Research Council rating scale for assessing paresis (grades: 0 = No contraction; 1 = Flicker or trace of contraction; 2 = Active movement with gravity eliminated; 3 = Active movement against gravity; 4 = Active movement against resistance; 5 = Normal strength). ^a^*n* = 22; ^b^*n* = 21; ^c^*n* = 20*.

Thirty-two neurologically healthy subjects served as controls and were matched to the patient group with respect to age (*M* = 54.6 years, *SD* = 7.7 years, range 42–70 years; *t*_(53)_ = −0.79, *p* = 0.432), right-handedness (i.e., laterality quotient (LQ) of the Edinburgh Handedness Inventory, Oldfield, 1971; *M* = 91.0, *SD* = 14.9; *t*_(50)_ = −1.63, *p* = 0.110), and gender (16 female; $\chi_{\left(1\right)}^{2}$ = 0.03, *p* = 0.874). None of the control subjects had a history of neurological or psychiatric diseases nor substance abuse or dependance. Moreover, both the stroke patients and the healthy controls constituted a representative sample in terms of education level and occupational profile. All participants had normal or corrected-to-normal visual acuity.

The study had been approved by the local Ethics Committee of the Medical Faculty of the University of Cologne and was performed in accordance with the ethical principles of the World Medical Association (Declaration of Helsinki; revised version, October, 2013). All participants provided written informed consent to participate in the study. The stroke patients additionally gave consent for using their clinical imaging data for lesion mapping.

### Apparatus and Stimuli

The experiment was programmed in Presentation^®^ (Neurobehavioral Systems, Inc.) and presented on a 15-inch computer screen.

**Figure 2:**
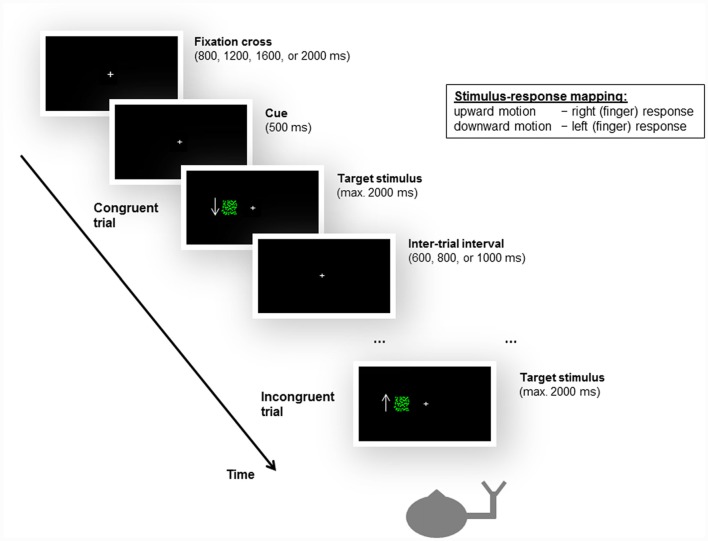
Illustration of the design and timing of the unimanual Simon task, as well as examples of a congruent and an incongruent trial. Participants were instructed to give left or right (finger) responses based on the motion direction of a moving dots stimulus, irrespective of the location (i.e., side of the fixation cross, left or right visual field) at which the stimulus appeared. For stimulus-response compatibility, left and right responses were mapped to the index and middle fingers of the same hand, i.e., either the left or the right hand. The stroke patients always responded with their ipsilesional hand. In the example shown here, the subject responds with the right hand, and upward motion is mapped to a right response and downward motion is mapped to a left response. Accordingly, when a downward-moving stimulus is presented in the left visual field, the trial is congruent; when an upward-moving stimulus appears in the left visual field, the trial is incongruent. Please note that the arrow was not presented to the participants during the experiment but is shown here to illustrate the motion direction of the stimulus.

As in the unimanual Simon task used by Arend et al. (2016), the peripheral stimuli consisted of a flow field of green moving dots against a black background. A white fixation cross was centrally displayed. Two patches of flow fields of upward and downward motion served as the task-relevant stimulus feature. The flow field density was set at 0.0075 dots/pixel^2^. The dots were randomly distributed within a square subtending 4° × 4° of visual angle. The boundaries of the square were not visible to the participants. The dots moved at a speed of 45 pixels per second. The patches of moving dots were displayed at one of two locations positioned such that their boundaries were 4° left or right of the fixation cross. The design and the timing of the experimental task are illustrated in Figure 2.

Participants were required to give left or right responses based on the motion direction of the moving dots (see below). Consequently, two trial types were defined by the correspondence between the spatial location at which the stimulus appeared and the side of response signaled by the motion direction of the stimulus. For *congruent trials*, the spatial location of the stimulus matched the side of response (e.g., an upward motion calling for a left response was presented on the left side of the fixation cross). For *incongruent trials*, there was a mismatch between the spatial location of the stimulus and the side of response (e.g., an upward motion calling for a left response was presented on the right side of the fixation cross).

### Procedure

Each participant was tested in a single session. The stroke patients were tested at the Department of Neurology of the University Hospital Cologne or at the Neurological Rehabilitation Centre Godeshöhe, Bonn. The healthy control subjects were tested at the Institute of Neuroscience and Medicine, Research Centre Jülich. All participants gave informed written consent and were comfortably seated at a table with the testing material and the computer screen for task presentation in front of them. Before the experimental task, stroke patients performed the above-described set of neuropsychological and clinical tests.

To avoid any confounding effects of contralesional paresis, stroke patients were instructed to respond with their ipsilesional hand. Accordingly, the responding hand is equivalent to the damaged hemisphere (left-hand response: *n* = 9, right-hand response: *n* = 14). Healthy control subjects were randomly assigned to respond either with their left (*n* = 17) or right hand (*n* = 15) to match stroke patients regarding the responding hand set-up ($\chi_{\left(1\right)}^{2}$ = 1.05, *p* = 0.305). The responding hand was always positioned to the right side (for the right-hand response group) or to the left side (for the left-hand response group) of the computer screen and thus the body midline.

For the Simon task, participants were instructed to give left or right responses corresponding to the motion direction of the stimulus on the screen, irrespective of the location (i.e., side of the fixation cross) at which the stimulus appeared. The stroke patients used a standard computer mouse to respond, the healthy controls used a LUMItouch response keypad. For stimulus-response compatibility in the current unimanual version of the Simon task, left and right responses were mapped to the index and middle fingers of the same, either of the left or of the right hand (Heister et al., 1987). In other words, for the participants responding with their right hand, left responses were given with the index finger and right responses were given with the middle finger. Accordingly, participants responding with their left hand gave left responses with the middle finger and right responses with the index finger.

The mapping between motion direction of the stimulus (downward and upward) and side of response (left and right) was counterbalanced across participants. For half of the participants, upward motion was mapped to a left response (i.e., middle finger of the left hand or index finger of the right hand) and downward motion was mapped to a right response (i.e., index finger of the left hand or middle finger of the right hand). For the other half of the participants, upward motion was mapped to a right response and downward motion was mapped to a left response.

Participants were required to respond as quickly and as accurately as possible. Response time (RT) in milliseconds was measured by the computer from stimulus presentation until the participant’s response. Errors, i.e., false responses, were also automatically recorded.

Each trial started with a central fixation cross that remained present throughout the task. The fixation cross subtended about 0.07° × 0.07° of visual angle and was presented for a variable period of 800, 1,200, 1,600, or 2,000 ms, after which a change in size (about 0.05° × 0.05° of visual angle) for 500 ms signaled the start of the trial. One of the two patches of moving dots (upward or downward) were then presented randomly either at the left or at the right side of the fixation cross and remained visible until participants made a response. If no response was given, the trial ended after 2,000 ms post-stimulus onset. After a variable inter-trial interval (ITI) of 600, 800, or 1,000 ms, the next trial started (see Figure 2).

Following a practice block of 20 trials for which participants received feedback (“Correct,” “Wrong,” or “No response”), each participant completed two experimental blocks, separated by a short break. Each experimental block contained 80 trials (i.e., 40 congruent trials and 40 incongruent trials) mixed within the block, resulting in a total of 160 experimental trials. The experiment took approximately 10–12 min to complete.

### Statistical Analysis

Statistical analyses were conducted using the software IBM SPSS Statistics (Statistical Package for the Social Sciences, Version 22, SPSS Inc., Chicago, IL, USA).

Independent-samples *t*-tests and chi-square analyses (for nominal variables) were used to compare means of demographic and clinical data between LH and RH stroke patients and between stroke patients and healthy controls. Repeated measures analyses of variance (ANOVAs) were used to analyze error rate and mean response times (RTs), separately. The error rate was calculated as the proportion of erroneous responses to the total number of trials, as a function of stimulus location and congruency. Error trials as well as RTs exceeding two standard deviations above or below the individual’s mean were discarded prior to the analysis to reduce skewness and prevent extreme RTs from influencing the mean of each participant (Ratcliff, 1993). The trimmed mean RTs for correct responses were then calculated as a function of stimulus location and congruency, irrespective of the preceding trial congruency.

The Kolmogorov-Smirnov tests (used to test for normality) on mean RTs of the stroke patients (*D*_(23)_ = 0.15, *p* = 0.191) and the healthy controls (*D*_(32)_ = 0.12, *p* = 0.200) were not significant, indicating that the RT data were normally distributed in both groups. However, the Levene’s test (used to test for homogeneity of variances between groups) on mean RTs was significant (*F*_(1,53)_ = 16.75, *p* < 0.001), indicating that the variances of RTs were significantly different for the stroke patients and the healthy controls. Early findings suggest that the *F*-statistic is a robust statistical model even when its assumptions are violated (Glass et al., 1972; Games, 1984). Accordingly, parametric tests were used. Nonetheless, to assure the reliability of the results with distribution-free statistics, non-parametric (*post hoc*) tests of the main results were additionally conducted.

The initial ANOVAs included *responding hand/lesioned hemisphere* as a between-subjects factor. However, there was no significant main effect of responding hand/lesioned hemisphere on error rate (*F*_(1,51)_ = 0.00, *p* = 0.988) or mean RT (*F*_(1,51)_ = 0.13, *p* = 0.717), and no interaction effect between responding hand/lesioned hemisphere and congruency or group approached statistical significance (all *p*-values > 0.180). Therefore, error rate and mean RT data were collapsed across responding hand/lesioned hemisphere for all further analyses. Thus, the final ANOVAs evaluated the effect of *group* (stroke patients vs. control subjects) as between-subject factor and *stimulus location* (ipsilesional/-lateral hemifield, contralesional/-lateral hemifield) and *stimulus-response congruency* (congruent, incongruent) as within-subject factors.

To further investigate the possible difference in the magnitude of the (overall) Simon effect between stroke patients and healthy controls, an additional Bayesian independent samples *t*-test (nondirectional, Cauchy prior = 0.707) was computed in JASP (version 0.8.1.1), a freely available statistical software (Rouder et al., 2009). The Bayes factor comparing the null hypothesis (H0) against the alternative hypothesis (H1; B_01_) is reported. The Bayes factor B_01_ reflects the evidence for H0 (i.e., the Simon effect is not different/is similar in the two groups) compared to H1 (i.e., the Simon effect is different in the two groups).

To assess post-error behavioral adjustments, the difference in mean RTs between (correct) post-error trials and all correct trials (both post-error and post-correct) was compared between the stroke patients and the healthy controls using an independent-samples *t*-test.

An analysis of the RT distributions was performed to determine the efficiency of selective response inhibition, based on the activation-suppression hypothesis (Ridderinkhof, 2002). For this purpose, correct RTs for each participant were rank-ordered separately for congruent and incongruent trials. Unfortunately, raw RT data were lost for one stroke patient. Each RT distribution was partitioned into four quantile bins of roughly equal size^4^ in each participant, and mean RTs were computed for each of the quartiles in each condition (i.e., congruent and incongruent). The Simon effect for each quartile was then obtained as the difference in mean RT for the incongruent and congruent conditions, and, averaged across subjects, plotted against the mean quartile RT (Vincentizing procedure; Ratcliff, 1979). A repeated measures ANOVA with *stimulus-response congruency* (congruent, incongruent) and *quartile* (Q1, Q2, Q3, Q4) as within-subject factors and *group* (stroke patients vs. control subjects) as between-subject factor was used to analyze the mean RTs in incongruent and congruent conditions as a function of response latency. To further examine a significant interaction effect with the factor group, separate ANOVAs were conducted for each group. Polynomial contrasts were then used to test for the trend in the RT difference between incongruent and congruent conditions across the RT distribution.

For all statistical analyses, a level of *p* < 0.05 was considered significant (with Bonferroni or Greenhouse-Geisser corrections, if applicable).

## Results

### Error Rate

Analysis of error rates revealed a significant main effect of congruency (*F*_(1,53)_ = 42.38, *p* < 0.001, $\eta_{\text{p}}^{2}$ = 0.44), reflecting that incongruent trials evoked overall more errors than congruent trials (6.8% vs. 2.9%). Moreover, the groups differed in overall error rate (*F*_(1,53)_ = 6.73, *p* = 0.012, $\eta_{\text{p}}^{2}$ = 0.11), with stroke patients making more errors compared to healthy controls (6.5% vs. 3.5%).

Importantly, the difference in error rate between incongruent and congruent conditions did not differ significantly between the groups (interaction group × congruency: *F*_(1,53)_ = 0.19, *p* = 0.665, $\eta_{\text{p}}^{2}$ = 0.004).

### Response Times

**Figure 3:**
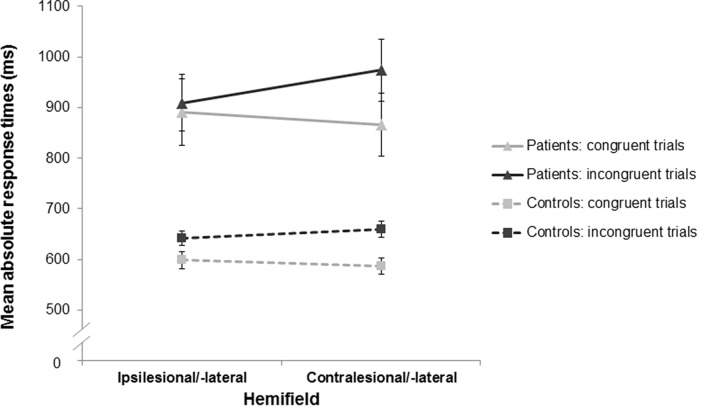
Mean response times (RTs) as a function of stimulus-response congruency and stimulus location for the stroke patients and the healthy controls. For both groups, RTs in the incongruent trials (dark gray) were longer than those in the congruent trials (light gray) indicating a significant Simon effect in the stroke patients (triangles, solid lines) and the healthy controls (squares, dashed lines). Furthermore, there was an asymmetry of the Simon effect in both groups with a more pronounced Simon effect in the contralesional/-lateral hemifield (compared to the ipsilesional/-lateral hemifield). Error bars indicate standard error of the mean (SEM).

Mean RTs for the stroke patients and healthy control subjects are presented in Figure 3.

There was a significant main effect of congruency (*F*_(1,53)_ = 79.70, *p* < 0.001, $\eta_{\text{p}}^{2}$ = 0.60): RTs for incongruent trials were longer than for congruent trials (772 ms vs. 712 ms), revealing the typical Simon effect. The main effect of group also reached significance (*F*_(1,53)_ = 28.93, *p* < 0.001, $\eta_{\text{p}}^{2}$ = 0.35), reflecting that stroke patients responded overall more slowly than healthy controls (910 ms vs. 622 ms). Considering the higher error rate in the stroke patients, their overall response slowing did probably not reflect a speed-accuracy trade-off.

Importantly, the RT difference between incongruent and congruent conditions did not differ significantly between the stroke patients and the healthy controls (interaction group × congruency: *F*_(1,53)_ = 0.16, *p* = 0.691, $\eta_{\text{p}}^{2}$ = 0.003). Indeed, this difference (i.e., the magnitude of the Simon effect) was similar in the two groups (63 ms for stroke patients; 58 ms for healthy controls). The similar magnitude of the Simon effect in the patient and control groups was also evident when mean RTs for congruent and incongruent conditions were adjusted for overall response latencies for each participant by means of proportion transformation (Faust et al., 1999; i.e., after taking into account overall group response speed differences; interaction group × congruency: *F*_(1,53)_ = 0.58, *p* = 0.450, $\eta_{\text{p}}^{2}$ = 0.01). Note that there was no significant difference in the magnitude of the Simon effect between the LH and RH stroke patients (64 ms for LH stroke patients, 62 ms for RH stroke patients; *t*_(21)_ = 0.08, *p* = 0.937, *d* = 0.03).

The Bayesian independent samples *t*-test on the magnitude of the (overall) Simon effect resulted in an estimated Bayes factor BF_01_ of 3.4, indicating substantial evidence for H0 (i.e., the hypothesis that the Simon effect is not different/is similar in the two groups).

Furthermore, there was a significant interaction effect of stimulus location and congruency (*F*_(1,53)_ = 7.42, *p* = 0.009, $\eta_{\text{p}}^{2}$ = 0.12). Planned comparisons revealed that RTs for incongruent trials were significantly longer in the contralesional/-lateral hemifield than in the ipsilesional/-lateral hemifield (791 ms vs. 754 ms; *t*_(54)_ = 2.91, *p* = 0.005, *d* = 0.79); whereas RTs for congruent trials did not show a significant difference between hemifields (704 ms vs. 721 ms; *t*_(54)_ = −1.45, *p* = 0.153, *d* = 0.40). Results indicated an asymmetry of the Simon effect that was smaller in the ipsilesional/-lateral hemifield (i.e., on the side of the responding hand; see Figure 3). Note that there was no significant difference of this asymmetry of the Simon effect between the stroke patients and the healthy controls (interaction group × stimulus location × congruency: *F*_(1,53)_ = 1.96, *p* = 0.167, $\eta_{\text{p}}^{2}$ = 0.04).

There was a significant post-error slowing in both stroke patients and healthy controls, which did not differ significantly between groups (77 ms for stroke patients, 100 ms for healthy controls; *t*_(48)_ = 0.77, *p* = 0.445, *d* = 0.22). Thus, the stroke patients and the healthy controls similarly adjusted their behavior after an error had occurred.

**Figure 4:**
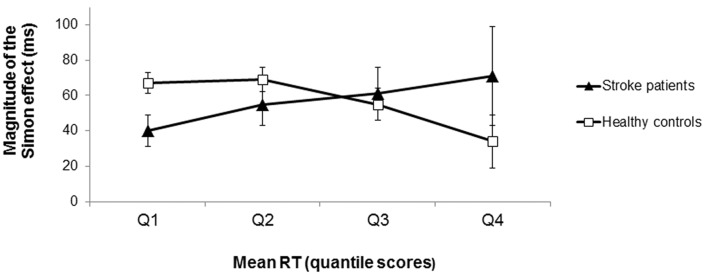
Magnitude of the Simon effect (difference in RTs between incongruent and congruent trials) as a function of response latency (in RT quantile scores) for the stroke patients and the healthy controls. For the healthy controls, the magnitude of the Simon effect decreased as RTs increased (squares). In contrast, the magnitude of the Simon effect remained stable across the RT distribution for the stroke patients (triangles) indicating less efficient selective response inhibition. Error bars indicate SEM.

The distributional analysis of RTs revealed a significant congruency × quartile × group interaction effect (*F*_(1.404, 73.024)_ = 4.34, *p* = 0.028, $\eta_{\text{p}}^{2}$ = 0.08; see Figure 4). Polynomial contrasts for each group separately showed that for the healthy controls the difference in RTs between incongruent and congruent conditions significantly decreased as RTs increased (interaction congruency × quartile: *F*_(1,31)_ = 5.82, *p* = 0.022, $\eta_{\text{p}}^{2}$ = 0.16 for the linear trend). In contrast, the RT difference between incongruent and congruent conditions did not significantly differ across the RT distribution for the stroke patients (interaction congruency × quartile: *F*_(1,21)_ = 1.47, *p* = 0.238, $\eta_{\text{p}}^{2}$ = 0.07 for the linear trend). Note that there was no significant difference between the LH and RH stroke patients concerning the course of the Simon effect as a function of response latency (*t*_(20)_ = 0.11, *p* = 0.913, *d* = 0.05). According to the activation-suppression hypothesis (De Jong et al., 1994), the stable difference in RTs between incongruent and congruent conditions (i.e., the stable Simon effect) across the RT distribution indicated less efficient selective inhibition in the patients with stroke-induced lesions of the striatum (in contrast to healthy controls).

Notably, the pattern of results could be replicated with non-parametric tests.

## Discussion

The aim of the present study was to investigate the putative contribution of the striatum (putamen and caudate nucleus) to the control of response interference. For that purpose, patients with unilateral striatal lesions (caused by stroke) and age-matched healthy controls performed a unimanual version of the Simon task. The magnitude of the Simon effect (reaction time difference between incongruent and congruent conditions) reflected the general ability to control response interference, and in combination with an analysis of the RT distributions the efficiency of selective inhibition of interfering responses.

Consistent with previous studies that successfully used unimanual Simon tasks (Heister et al., 1987; Wiegand and Wascher, 2007; Arend et al., 2016), both stroke patients and healthy controls exhibited a significant Simon effect. Most importantly, the magnitude of the Simon effect did not differ significantly between the stroke patients and the healthy controls (63 ms for stroke patients; 58 ms for healthy controls), even after taking into account the differences in overall response latencies between the two groups. Thus, stroke patients—despite their unilateral lesions of the striatum—showed a similar ability to control response interference as healthy subjects, independent of the lesioned hemisphere. However, by considering the temporal dynamics of the processes underlying response interference control, stroke patients showed less efficient selective inhibition of interfering responses compared to healthy controls, independent of the lesioned hemisphere.

At first glance, the preserved Simon effect in the stroke patients with unilateral lesions of the striatum may contrast with previous clinical studies revealing reduced control of response interference in a Simon task in patients suffering from neurodegenerative diseases involving the striatum (i.e., PD, or HD; Georgiou et al., 1995; Praamstra and Plat, 2001; Fielding et al., 2005). On the other hand, the current results are in line with other clinical studies that used the Simon task in patients with PD and HD and showed that the control of response interference was preserved in these patients despite (fronto-) striatal neurodegeneration (Brown et al., 1993; Cope et al., 1996; Georgiou-Karistianis et al., 2007; Schmiedt-Fehr et al., 2007). Moreover, a previous clinical study in PD patients likewise reported preserved response interference control but reduced efficiency of selective inhibition in a Simon task by applying distributional analyses (Wylie et al., 2010).

The current results of the distributional analysis in the stroke patients with unilateral striatal lesions are consistent with the assumption that less efficient selective inhibition would manifest in a stable or rather increasing Simon effect across the RT distribution. This assumption is based upon both theoretical frameworks of the selective inhibitory process (and its interpretation) in the Simon task (Ridderinkhof, 2002) and many (Ridderinkhof et al., 2005; Castel et al., 2007; Juncos-Rabadán et al., 2008) albeit not all previous patient studies (Wylie et al., 2010).

The most parsimonious explanation for the preserved (general) ability to control response interference in the current sample of stroke patients with unilateral striatal lesions is that the functions of the lesioned striatum were compensated for by the contralesional striatum and/or by (frontal) cortical regions. This notion is corroborated by imaging studies in healthy participants showing that the Simon task activated the striatum bilaterally in addition to frontal areas, including anterior cingulate and lateral prefrontal cortices (Nee et al., 2007; Zhang et al., 2017).

With respect to previous studies in stroke patients with unilateral striatal lesions, there are currently only three studies available that investigated cognitive control processes, namely cognitive flexibility (Cools et al., 2006; Yehene et al., 2008) and response inhibition (Rieger et al., 2003). Using task switching and the stop-signal task, these studies revealed impaired flexible and inhibitory control functions in patients suffering from unilateral strokes involving the striatum, while the current study revealed no relevant deficit in another cognitive control function, namely the general ability to control response interference. These apparently divergent results may depend on specific task demands. While sharing a common need to control prepotent response tendencies (Aron, 2011), the cognitive control process assessed by the Simon task (i.e., response interference control) is subtly different from that required for (global) response inhibition (assessed by stop-signal or go/no-go tasks; Egner et al., 2007) or for cognitive flexibility (assessed by task-switching or set-shifting tasks; Diamond, 2013).

One could argue that the sample size of the current study may have been too small to reliably detect deficits in the (general) ability to control response interference in stroke patients with striatal lesions. However, note that the Bayesian statistics revealed a Bayes factor BF_01_ of 3.4, indicating substantial evidence for the hypothesis H0 that the (overall) Simon effect is not different between the patient and control groups. Moreover, the sample sizes in the studies that reported impaired response inhibition (Rieger et al., 2003) and cognitive flexibility (Cools et al., 2006; Yehene et al., 2008) in stroke patients with striatal lesions were clearly smaller (6–8 patients) than the current patient sample size (*n* = 23).

Taken together, these previous findings and our current results suggest a specific role of the striatum in cognitive control processes, namely in (global) response inhibition (and cognitive flexibility) as well as in the efficiency of selective inhibition of interfering responses (engaged in response interference control). The above-mentioned studies and the current study also indicate that it is important to precisely characterize the cognitive control process under investigation when trying to elucidate the neural basis of cognitive control.

The differential contribution of the striatum to some (response inhibition and cognitive flexibility), but not other (general ability of response interference control) cognitive control processes may be grounded in the involvement of different subparts of the striatum in the diverse fronto-striatal networks related to cognitive control (Middleton and Strick, 2000; Utter and Basso, 2008). In this vein, the striatum could be involved in cognitive control processes in the context of eye movements (e.g., by connections with the frontal eye fields, FEFs) rather than in spatial coding *per se* (i.e., how response and spatial codes are represented in the context of the Simon task; Henik et al., 1994; Van der Stigchel et al., 2010). Note that we asked our subjects to centrally fixate during the Simon task, while other studies allowed eye movements or even used saccades to measure response latencies (Fielding et al., 2005). Therefore, it is conceivable that stroke patients with striatal involvement may exhibit impaired control of response interference in tasks requiring eye movement responses, but performed relatively unimpaired in the current task requiring unimanual finger responses. This hypothesis of an effector-dependent involvement of the striatum in the control of response interference warrants further investigation.

In addition, the current results of the unimanual version of the Simon task have implications for theoretical accounts of spatial coding in the Simon task. For both groups (stroke patients and healthy controls), the Simon effect was smaller in the ipsilesional/-lateral hemifield. This pattern of results was also observed in (right-handed) young healthy controls (Arend et al., 2016; see also Supplementary Figure S2), and is in line with the grouping model (Adam et al., 2003) that accounts for the effects of the location of the stimulus and that of the responding hand on the Simon effect in *unimanual* experimental setups. The grouping model assumes that pre-attentive grouping processes may pose an advantage when the stimulus activates two associated responses. In the unimanual version of the Simon task used here, left and right responses were given with the index and middle fingers of the same hand and therefore the two fingers were part of the same response unit. Following the grouping model, if a participant responded with the right hand, the presentation of the stimulus in the right visual field (i.e., ipsilateral hemifield) probably activated both the index and middle fingers of the right hand because they belong to the same response unit (i.e., the right hand). Consequently, the mismatch between the spatial location of the stimulus and the side of response in incongruent conditions should be reduced on the side of the responding hand, which in turn should reduce the difference between incongruent and congruent conditions (i.e., the Simon effect; Arend et al., 2016), as could be observed in the current study.

## Conclusion

When adopting a unimanual Simon task, stroke patients with unilateral lesions of the striatum showed preserved yet less efficient control of response interference. Moreover, the finding of a reduced Simon effect in the ipsilesional/-lateral hemifield—in both stroke patients and healthy controls—supports the grouping model (Adam et al., 2003; Arend et al., 2016).

## Author Contributions

CS: analysis and interpretation of the data; conception, writing and revision of the manuscript. DT: study design; acquisition and analysis of the data; lesion mapping; critical revision of the manuscript. IA and SV: study concept and design; analysis of the data; critical revision of the manuscript. AD: lesion mapping; critical revision of the manuscript. JS and HK: acquisition of the data; critical revision of the manuscript. GF and AH: study concept and supervision; critical revision of the manuscript. PW: study concept, design and supervision; critical revision of the manuscript. All the authors have approved the final version of the manuscript and agree to be accountable for all aspects of the work.

## Conflict of Interest Statement

The authors declare that the research was conducted in the absence of any commercial or financial relationships that could be construed as a potential conflict of interest.

## Footnotes

^1^http://people.cas.sc.edu/rorden/mricron/index.html

^2^http://www.nmr.mgh.harvard.edu/

^3^https://fsl.fmrib.ox.ac.uk/fsl/

^4^The number of bins was chosen to provide a reasonable estimate of bin values of about 20 trials for congruent and incongruent conditions per quartile (Ratcliff, 1979).
