# Supplementary material for: Preserved but Less Efficient Control of Response Interference After Unilateral Lesions of the Striatum
Source: Front Hum Neurosci. 2018 Oct 16;12:414. doi: 10.3389/fnhum.2018.00414 (PMC6232767; doi:10.3389/fnhum.2018.00414)
Supplement: Supplementary file 1 [file Data_Sheet_1.PDF]

Supplementary Material

Preserved but less efficient control of response interference after unilateral lesions of the striatum

Claudia C. Schmidt*, David C. Timpert, Isabel Arend, Simone Vossel, Anna Dovern, Jochen Saliger, Hans Karbe, Gereon R. Fink, Avishai Henik & Peter H. Weiss

*** Correspondence:** Claudia Schmidt: c.schmidt@fz-juelich.de

1 Supplementary Figures

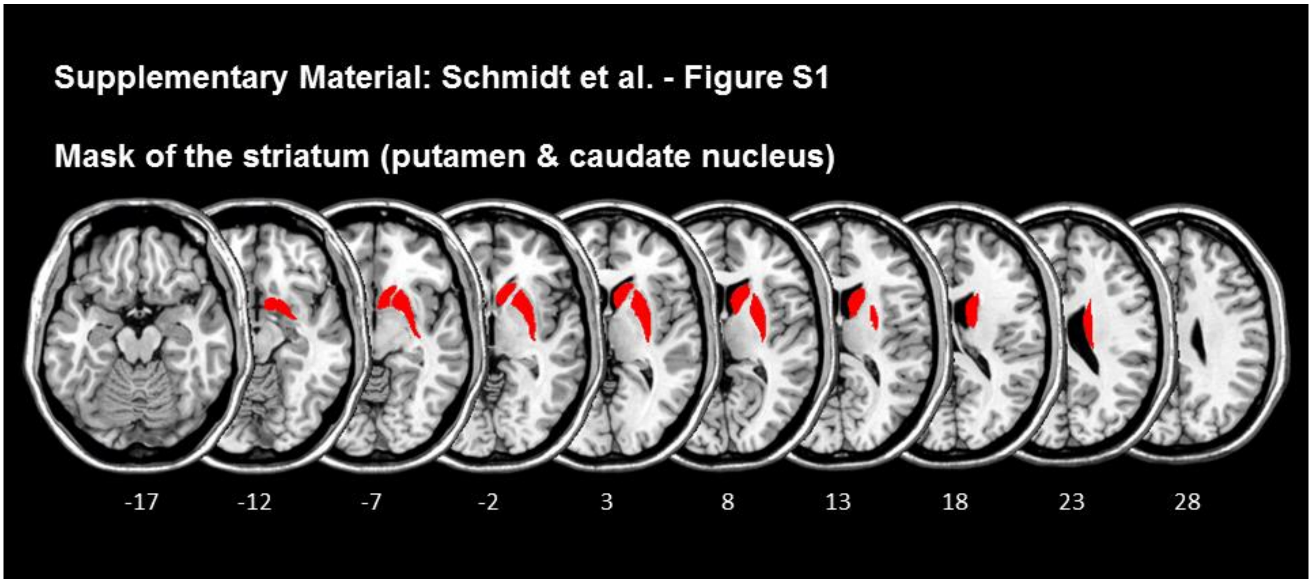

Supplementary Figure S1. Visual depiction of a mask of the striatum (putamen and caudate nucleus) derived from the Harvard-Oxford atlas of cortical and subcortical structures provided by the Harvard Center for Morphometric Analysis (http://www.cma.mgh.harvard.edu/) and distributed with FSL (https://fsl.fmrib.ox.ac.uk/fsl/). This mask was used to verify the involvement of the striatum in the current sample of stroke patients.

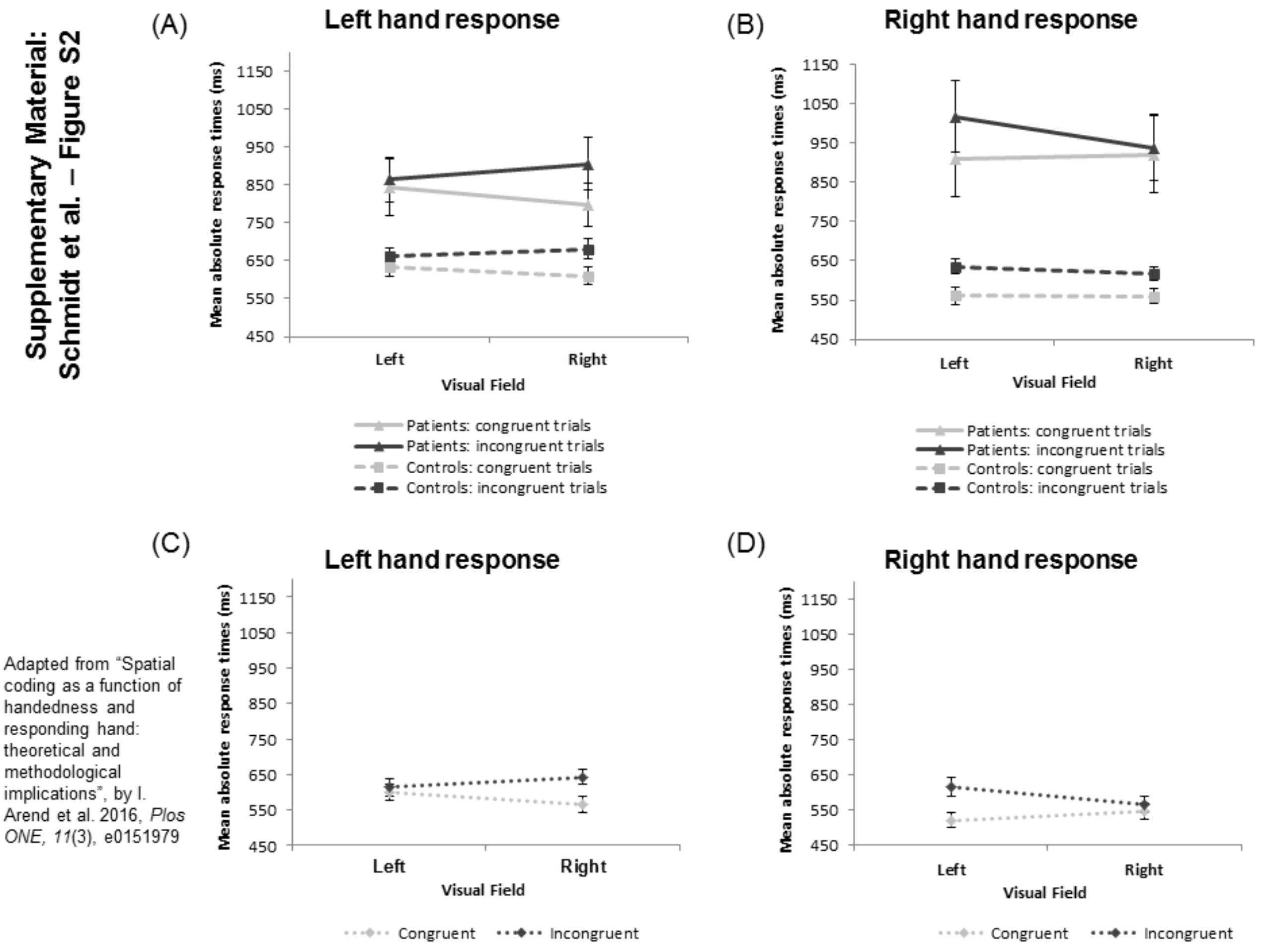

Supplementary Figure S2. Mean response times (RTs) as a function of stimulus-response congruency, stimulus location, and responding hand for the stroke patients and the age-matched older healthy controls (A, B) as well as for young healthy controls (C, D)

For all three groups, RTs in the incongruent trials (dark grey) were longer than those in the congruent trials (light grey) indicating a significant Simon effect in stroke patients (triangles, solid lines), older healthy controls (squares, dashed lines), and (right-handed) younger healthy controls (diamonds, dotted lines; data from Arend et al., 2016). Furthermore, there was an asymmetry of the Simon effect in all three groups with a more pronounced Simon effect in the contralesional/-lateral hemifield (compared to the ipsilesional/-lateral hemifield).

For the stroke patients and the older healthy controls alike, there was an additional significant interaction effect between responding hand/lesioned hemisphere and stimulus location [$F(1,51) = 4.46$, $p = .040$, $\eta_p^2 = .08$]. Planned comparisons revealed that participants responding with the right hand (B) responded slower to stimuli presented in the contralesional/-lateral compared to the ipsilesional/-lateral hemifield [774ms vs. 753ms; $t(28) = -2.39$, $p = .024$, $d = 0.9$], whereas participants responding with the left hand (A) did not show a significant difference in RTs between hemifields [717ms vs. 719ms; $t(25) = 0.39$, $p = .699$, $d = 0.2$]. Error bars indicate standard error of the mean (SEM).
